# Indiana, Illinois and Kentucky Tri-State Medical Society

**Published:** 1879-01

**Authors:** 


### ARTICLE X.

### INDIANA, ILLINOIS AND KENTUCKY TRI-STATE MEDICAL SOCIETY.

The fourth annual meeting was held in the Hall of Representatives, in the city of Springfield, Ill., Nov. 13, 14 and 15, 1878.

The society was called to order at 11 a. m., by the president, Dr. James F. Hibbard, of Richmond, Ind. The session was opened with prayer by Rev. G. H. Fullerton.

Judge J. H. Matheny, on behalf of the citizens of the State and city, made a brief speech of welcome to the Society, paying a high tribute to the pioneers of our profession.

Dr. Theophilus Parvin, of Indianapolis, responded to the judge, in a brief and eloquent speech.

Dr. B. M. Griffith, chairman of the Committee of Arrangements, submitted his report, which was adopted. The report gave the order of exercises and the programme for each day; it also stated: "This society has no ethical matters to discuss, not proper in any medical organization, its sole object being the reading and discussion of scientific papers and cultivating professional sociability." And yet, none are received to membership who are not members of some local society auxiliary to the American Medical Association.

After calling the various committees, the society adjourned to 2:30 p. m.

AFTERNOON.

Dr. Hibbard, the president, delivered the annual address, which treated at some length of, the functions of the nerves and of the brain.

Dr. David Prince, of Jacksonville, read a paper on the "Metric System." The reader gave illustrations upon the blackboard, and explained the practical advantages of the system.

Dr. J. M. Holloway, of Louisville, read a paper on "Muscular Rigidity—a Symptom, Cause or Result of Disease." The doctor reported several cases in illustration.

Adjourned to 7:30 p. m.

EVENING.

Dr. J. Adams Allen, of Chicago, delivered a public address on the "Nervous System."

Dr. J. W. Singleton, of Paducah, Ky., read a paper on the "Medical Heroism" of 1878. The doctor recounted the sacrifices made by the members of the profession in the great scourge of the year. It is to be hoped that the entire profession will pay the tribute due to the memory of "our fallen heroes." Who can say that the bravery of Cook, Reuter, Rodgers, Saup, Easley, Woolfok and hosts of others, who Christ-like gave their lives for men, has ever been excelled?

Adjourned to 9 a. m.

NOV. 14.—SECOND DAY.

Dr. T. Parvin, Indianapolis, read an interesting paper on "Perineoplasty." The doctor reported several cases of perineal rupture treated by this method.

"Modes of Administering Mercury in Syphilis," was the subject of a paper by Dr. J. W. Thompson, of Paducah, Ky.

Dr. J. S. Hodgen, of St. Louis, read an exhaustive paper on "Section of the Superior Maxillary Nerve." The various surgical operations resorted to in the treatment were presented and clearly illustrated by suitable drawings.

AFTERNOON.

Dr. Thos. F. Rumbold, St. Louis, read a paper on the "Treatment of Scarlatina," dwelling at some length on mechanical therapeutics.

Dr. J. S. Jewell, of Chicago, reported on recent researches in the anatomy of the spinal cord.

Officers for the ensuing year were elected as follows: President, Dr. J. A. Ireland, Louisville, Ky; 1st Vice-President, Dr. J. W. Compton, Indiana; 2d Vice-President, Dr. B. M. Griffith, Illinois; 3d Vice-President, Dr. J. M. Holloway, Ky. Secretary, Dr. G. W. Burtal, Mitchell, Ind.; Assistant Secretary, Dr. H. H. Dening, Pana, Ills.; Treasurer, Dr. F. W. Beard, Vincennes, Ind.

*Chairmen of Sections.*—Surgery, Dr. J. M. Keller, Hot Springs, Ark.; Practical Medicine, Dr. S. H. Charlton, Seymour, Ind.; Obstetrics, Dr. H. B. Buck, Springfield, Ills.; Gynæcology, Dr. J. W. Singleton, Paducah, Ky.; State Medicine, Dr. Thad. M. Stevens, Indianapolis, Ind.; Pathology and Microscopy, Dr. S. L. Mathews, Springfield, Ills.

*Chairman of Committee of Arrangements for* 1879.—Dr. A. M. Owen, Evansville, Ind.

Time of next meeting, first Tuesday in November, 1879; place, Evansville, Ind.

A paper on "Pelvic Cellulitis," was read by Dr. J. A. Ireland, Louisville.

"Finger Stumps" formed the topic of the next paper, by Dr. H. C. Fairbrother, East St. Louis.

Adjourned to 7:30 p. m.

### EVENING.

Dr. E. H. Gregory, of St. Louis, read a paper, entitled, "What is man?"

Adjourned.

By special invitation, the Society attended a reception at the Executive Mansion. His Excellency the Governor, Mrs. Cullom and their daughters, gave the society a welcome that will be a pleasant recollection to every member in attendance.

### NOV. 15.—THIRD DAY.

Dr. Thad. M. Stevens' paper, "Some Thoughts on Certain Medico-Legal Questions," was read by title.

Dr. Sarah H. Stevenson, of Chicago, read a paper entitled, "Education of the Senses."

Dr. J. M. Keller, of Hot Springs, Ark., read an interesting paper on "Yellow Fever." The Doctor believes that "national quarantine" is the remedy for this terrible scourge.

Dr. J. W. Compton, of Evansville, followed, in the afternoon session, with a paper on the same subject. He believed the poison was solid, but not of any form of life; that it attached itself to walls and exposed surfaces. He believed the only preventive was a strict quarantine, and a stringent local enforcement of sanitary measures.

A Committee on Quarantine was appointed by the chair, to report at the next annual meeting, consisting of Dr. J. M. Keller, Hot Springs; Dr. D. H. Chase, Louisville, Ills.; and Dr. E. S. McIntire, Mitchell, Ind.

The papers, after discussion, were all referred to the Committee on Publication.

A number of papers were read by title and referred.

Society adjourned to 8 p. m.

According to previous notice, the work of the Society was to conclude with a microscopic exhibition, under the management of S. L. Mathews, chairman of section on Pathology and Microscopy. There were present thirty-five instruments, and objects of every description. Jacksonville Microscopical Society, fourteen in number; the first was a monocular, in charge of Dr. H. K. Jones; object, the circulation of the blood in a frog's foot. Dr. G. V. Black, with his binocular, exhibited the circulation of the blood in the lung of the frog. Miss Fuller exhibited ocean shells; Dr. Pitner, viscera of rabbit; Mrs. Mulligan, pollen of lily; A. E. Prince, fly and spider's eye; Miss Rhodes, living hydra. Several other instruments, in charge of Drs. Frost, Winslow, Corwin and others. Dr. Mathews manipulated nine instruments, namely: the Henry Crouch, great binocular, the intermediate, the student's monocular, the educational monocular, the queen binocular and monocular, and two of Bullock's monoculars, of Chicago. Dr. M. exhibited trichinæ from pork; itch mite, human blood, deep sea soundings, etc. Close by was Dr. V. S. Lindsay, with his Hartnack monocular, and about fifty pathologico-anatomical specimens. Near him was the writer, with Ross monocular, specimens all pathological. Next came the tables of the Chicago society, two instruments in charge of Dr. Danforth, who also exhibited the circulation in the frog's foot, lung and the action of the heart. J. G. Langguth had ten instruments, with every variety of mounted objects; the most interesting was a living trichina.

With a vote of thanks to Governor Cullom, the citizens of the State and city, the society adjourned, to meet in the city of Evansville, Ind., first Tuesday in November, 1879. B.

---

A CHEERFUL PROSPECT FOR PHYSICIANS IN CHICAGO.—The Chicago organ of one of our religious "denominations," urges its readers to promote the social element in the church by following the example of a family where there was an informal social gathering. "No refreshments were served, but at one corner was a table with cold water, popped corn, raisins, walnuts and doughnuts."

There is a demand for ostrich pepsin in the retail drug trade; and it is understood that the homœopathists are preparing a tenth decimal trituration of "cold water, popped corn, raisins, walnuts and doughnuts."

